# 876. Suggested Empiric Therapy for CoNS Identified by BioFire BCID2 – Making Mountains Out of Molehills?

**DOI:** 10.1093/ofid/ofac492.069

**Published:** 2022-12-15

**Authors:** HaYoung Ryu, Diana Yu, James S Lewis

**Affiliations:** Oregon Health & Science University Hospital and Clinics, Portland, OR; Doernbecher Children's Hospital/OHSU Healthcare, Portland, Oregon; Oregon Health and Science University, Portland, Oregon

## Abstract

**Background:**

Coagulase-negative Staphylococci (CoNS), including *mecA-*positive *Staphylococcus epidermidis* (MRSE), are among the bacteria identified by BioFire blood culture identification (BCID2). After transition to BCID2 on 31 March 2021, our institution began providing a suggested empiric antimicrobial therapy (EAT) comment with rapid diagnostic results, whereas no suggestions were given with the prior platform. Limited data exist on the impact of EAT suggestions on antibiotic use when likely contaminants such as CoNS are identified.

**Methods:**

This was a single center, observational, pre- and post-intervention study of patients aged ≥ 18 years with CoNS in the blood between 1 April 2020 and 30 September 2021. Patients with compromised immunity, intravenous drug use, polymicrobial cultures, or MRSE-active therapy use for a documented infection were excluded. Primary outcome was the rate of initiation or discontinuation of MRSE-active agent in response to the rapid diagnostic result. Secondary outcome was healthcare resource utilization such as Infectious Diseases consultation and delayed discharge.

**Results:**

A total of 174 patients were included (pre-BCID2, n=93; post-BCID2, n=81). For all CoNS rapid diagnostic results, no significant difference was noted in MRSE-active agent initiation (33.3% vs. 37.0%, p=.90) and discontinuation (61.3% vs. 48.4%, p=.31). A subgroup analysis of *S. epidermidis* was performed with 64 and 46 patients in pre- and post-BCID2 groups, respectively; in the latter, BCID2 identified 31/46 (67.4%) as MRSE. In this subgroup, empiric MRSE-active agent was significantly less likely to be discontinued if EAT suggestion was reported with results (68.4% vs. 31.2%, p=.03). This change was not seen if Staphylococcus species were identified as the suggestion was to withhold EAT. There was no difference in healthcare resource utilization.

**Conclusion:**

Providing EAT suggestion with BCID2 result for *S. epidermidis* unexpectedly encouraged continuing empiric MRSE-active agent for likely contaminants. In contrast, suggestion to withhold treatment had no impact on EAT use for other CoNS. The reason for this difference is unclear, but may be a result of the highlighted methicillin resistance in the BCID2 result reporting which is specific to *S. epidermidis*.

**Disclosures:**

**James S. Lewis, PharmD, FIDSA**, Cidara: Advisor/Consultant|Merck: Advisor/Consultant|SeLux Diagnostics: Advisor/Consultant.

